# Successful Management Of Tumor‐Induced Osteomalacia with Radiofrequency Ablation: A Case Series

**DOI:** 10.1002/jbm4.10178

**Published:** 2019-02-28

**Authors:** Sunil Kumar Mishra, Mohammad Shafi Kuchay, Ishita Barat Sen, Arpit Garg, Sanjay Saran Baijal, Ambrish Mithal

**Affiliations:** ^1^ Division of Endocrinology and Diabetes Medanta‐The Medicity Hospital Gurugram India; ^2^ Department of Nuclear Medicine Fortis Memorial Research Institute Gurugram India; ^3^ Department of Radiology Medanta‐The Medicity Hospital Gurugram India

**Keywords:** ONCOGENIC OSTEOMALACIA, TUMOR‐INDUCED OSTEOMALACIA, RADIOFREQUENCY ABLATION, PHOSPHATURIA, HYPOPHOSPHATEMIA, RENAL PHOSPHATE WASTING

## Abstract

Tumor‐induced osteomalacia (TIO) is a curable condition when the tumor is correctly located and completely removed. These tumors are, however, small and located in regions that make surgical removal difficult and sometimes risky in some patients. Experience of radiofrequency ablation (RFA) in the management of TIO is limited. We describe 3 patients with TIO who were treated in our hospital with RFA. They had suspected lesions in surgically difficult locations and were subjected to single sessions of RFA. The response was documented in terms of improvement in symptoms, normalization of hypophosphatemia and hyperphosphaturia, and disappearance of uptake on follow‐up Ga^68^ DOTANOC PET/CT imaging. All 3 patients had a clinical and biochemical profile consistent with TIO. The first patient (patient 1) had an intensely Ga^68^ DOTANOC avid lesion involving the roof of right acetabulum. The second patient (patient 2) had a Ga^68^ DOTANOC avid intramuscular lesion in left pectineus muscle and the third patient (patient 3) had a Ga^68^ DOTANOC avid expansile osteolytic lesion involving the angle and ramus of right mandible. All 3 patients achieved complete biochemical as well as clinical remission with single sessions of RFA. Six months after the procedure, Ga^68^ DOTANOC imaging revealed the absence of uptake at the previous sites, corroborating with the clinical improvement and normalization of hypophosphatemia and hyperphosphaturia. In conclusion, although surgical resection is the standard of care, RFA can be used successfully for treating patients with TIO. It can be an effective, less invasive, and safe modality of treatment in those patients where resection of the lesion is not possible because of inaccessible anatomical location or comorbidity that prohibits surgery. © 2019 The Authors. *JBMR Plus* published by Wiley Periodicals, Inc. on behalf of American Society for Bone and Mineral Research.

## Introduction

Tumor‐induced osteomalacia (TIO) is an interesting paraneoplastic syndrome of acquired hypophosphatemic osteomalacia. It is caused by a phosphate and vitamin D regulating hormone known as fibroblast growth factor‐23 (FGF 23).^1^, ^2^ FGF‐23 downregulates sodium‐phosphate cotransporters (NaPi‐2a/NaPi‐2c) and 25‐hydroxyvitamin D 1‐alpha‐hydroxylase in the proximal renal tubules. This results in increased phosphate excretion and to a lesser extent decreased intestinal phosphate absorption.^3^ FGF‐23 also upregulates the expression of vitamin D 24‐hydroxylase, a mitochondrial enzyme responsible for inactivating vitamin D metabolites.^4^ Therefore, FGF‐23 appears to serve the primary purpose of maintaining phosphorus homeostasis by enhancing urinary phosphate excretion, while decreasing intestinal phosphorus absorption through lower circulating serum concentrations of 1,25 dihydroxyvitamin D (1,25(OH)_2_D). FGF‐23 is usually elaborated by mesenchymal tumors. The localization of these tumors is challenging because they can be present virtually anywhere in the body and are often very small.^5^ Several functional imaging modalities (^111^In‐pentetreotide single‐photon emission CT [Octreoscan‐ SPECT/CT], fluoro‐deoxyglucose‐PET/CT [^18^F FDG‐PET/CT] scan, ^68^Ga‐DOTATATE PET/CT, etc.) have been utilized for localizing these small tumors. Ga^68^ DOTANOC PET/CT scan is said to have the greatest sensitivity and specificity for detecting small mesenchymal lesions, giving rise to TIO.^6^, ^7^ The standard treatment of TIO is surgical excision of the tumor. The patients with TIO can be cured completely if the tumor is localized correctly and excised adequately.^8^ However, these tumors are often present in difficult locations, deep in the bones or close to joints. In such situations, surgery might endanger the adjacent joints or disproportionate tissue injury might occur in an attempt to access the tumor. Such cases require less invasive modalities of treatment like radiofrequency ablation (RFA). We present 3 patients with TIO where RFA was used successfully.

## Case Reports

### Patient 1

A 43‐year‐old male presented with a 5‐year history of diffuse bony pains, pain in ribs, and proximal muscle weakness. The pain was insidious in onset but progressively increased in severity. For the last 1 month, he was unable to walk because of muscular weakness. There was no similar illness in his family. He had received treatment for bony pains in the past. His medications included calcium (elemental calcium 500 mg/d), phosphate powder 3 g/d, cholecalciferol 2000 IU/d, and various analgesics. He had partial response to these medications clinically, but his serum phosphate level had never normalized. Biochemical investigations of the patient are given in Table 1. TIO was defined by hypophosphatemia, elevated serum FGF‐23, low 1,25(OH)_2_D, and decreased percentage of tubular reabsorption of phosphate (%TRP). %TRP was calculated according to the formula: 100 × (1–[urine phosphate/urine creatinine] × [serum creatinine/serum phosphate]). Radionuclide bone scan (after intravenous administration of 20 mCi of Tc‐99m MDP) revealed insufficiency fractures in multiple ribs, bilateral femoral neck, sacrum, and bilateral distal tibia. The overall scintigraphic findings suggested metabolic bone disease, possibly osteomalacia. Magnetic resonance imaging (MRI) revealed fracture of femoral neck bilaterally in subcapital region. Gallium‐68 DOTANOC positron emission tomography (PET) with contrast‐enhanced CT scan was performed. It suggested an intensely Ga^68^ DOTANOC avid lesion involving the roof of right acetabulum, measuring about 1.1 × 0.9 × 1.4 cm. The scan also suggested insufficiency fractures involving necks of bilateral femorii and bilateral sacral ala.

**Table 1:** Biochemical Evaluation of Patients 1 to 3, Pre‐RFA and Post‐RFA

	Patient 1		Patient 2		Patient 3		
Parameters	Pre‐RFA	Post‐RFA (day 14)	Pre‐RFA	Post‐RFA (day 7)	Pre‐RFA	Post‐RFA (day 14)	Normal range
Albumin‐corrected total calcium (mg/dL)	10.2, 10.3	9.3, 9.8	10.0, 9.7	9.4, 9.6	9.9, 9.5	9.5, 9.6	8.5–10.4
Serum phosphorus (mg/dL)	1.3, 1.5	2.8, 3.0	1.4, 1.5	3.1, 3.4	2.0, 1.8	3.1, 3.3	2.4‐4.4
Serum ALP (U/L)	279	286	705	221	212	272	30–120
Serum 25(OH)D (ng/mL)	19.3	—	62.6	54.2	49.6	52	50–250
1,25(OH)_2_D (pmol/L)	20.0	—	38.2	—	50.1	–	47.7–190.3
Intact PTH (pg/mL)	52.7	81.6	52.7	61.0	212.0	41	14.0–72.0
TRP (%)^a^	81	90	80	91	75	90	88–95
TMP/GFR (mg/dL)	1.5	3.0	1.3	3.2	1.1	2.9	2.8–4.4
c‐FGF‐23 (RU/mL)^b^	453.0	—	960.0	135.0	101.8	—	<180
Site of lesion	Roof of right acetabulum		Left pectineus muscle		Angle and ramus of right mandible		—
Duration of follow‐up after RFA (months)	24		24		10		—

RFA = radiofrequency ablation; ALP= alkaline phosphatase; 25(OH)D = 25 hydroxyvitamin D; 1,25(OH)_2_D = 1,25 dihydroxyvitamin D; PTH = parathyroid hormone; TRP = tubular reabsorption of phosphate; TMP/GFR = ratio of tubular maximum reabsorption of phosphate to glomerular filtration rate; c‐FGF‐23 = C‐terminal fibroblast growth factor‐23.

^a^ TRP (%)  is calculated according to the formula: 100 × (1 − [urine phosphate/urine creatinine] × [serum creatinine/serum phosphate]).

^b^ Carboxy‐terminal FGF‐23 (ELISA, Immutopics, Inc., San Clemente, CA, USA).

**Figure 1:**
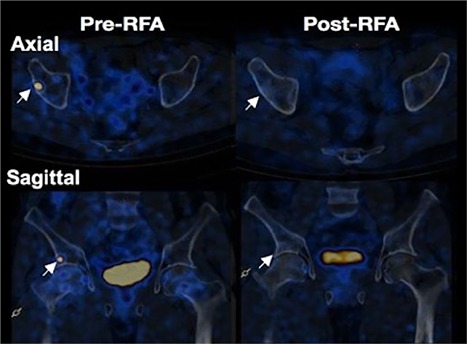
Pre‐ and post‐RFA Ga^68^ DOTANOC PET/CT scans of patient 1. Axial and sagittal views of the lesion in patient 1. Post‐RFA images demonstrate disappearance of the uptake at the lesion site. Arrows indicate the lesion.

Because the tumor was present at a surgically difficult location in the right acetabulum, surgical removal of the lesion required right hip replacement, after discussion with the orthopedics team, for which the patient was reluctant. Alternatively, the patient was offered a single session of RFA, after discussion with the interventional radiology team. A biopsy of the lesion was performed before ablation. However, it revealed only scanty tissue that was insufficient for opinion. It should be noted that biopsy carries a risk of seeding and probably an increased risk of recurrence later on. Regional venous sampling could be an alternative. In our case, we did not attempt regional venous sampling. Serum phosphorus and renal tubular reabsorption of phosphate returned to normal when evaluated 2 weeks after the RFA. His symptoms resolved significantly within 2 months and he was able to walk. Six months after RFA, a repeated Ga^68^ DOTANOC PET/CT scan revealed absence of uptake in the previous acetabular lesion (Fig. 1). He was asymptomatic and has normal serum phosphorus 2 years after RFA treatment.

### Patient 2

A 67‐year‐old male with type 2 diabetes mellitus and primary hypothyroidism presented with a 3‐year history of progressive muscular weakness and widespread musculoskeletal pain that severely impacted his mobility and quality of life. His medication before RFA included calcium, vitamin D, and analgesics. There was no relief of his symptoms. He has no family history of a similar illness. Biochemical analyses revealed persistent hypophosphatemia, decreased %TRP, elevated serum FGF‐23, and low 1,25(OH)_2_D, suggesting TIO (Table 1, patient 2). Skeletal survey revealed multiple pseudo‐fractures, suggesting metabolic bone disease. Functional imaging by Ga^68^ DOTANOC PET/CT scan showed a 2.5 × 2.1 × 2.1 cm intramuscular lesion with peripheral enhancement in left pectineus muscle. Because the patient was reluctant for surgery for personal reasons, a single session of RFA of the pectineal lesion was offered in consultation with the interventional radiology team. One week after RFA, his serum phosphorus was normalized. Six months after RFA, his symptoms had resolved completely. The patient remained asymptomatic with normal serum phosphorus levels for 2 years after RFA treatment.

### Patient 3

**Figure 2:**
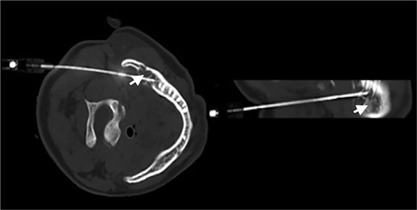
Electrode of RFA in situ, expansile lesion in the angle of right mandible, as indicated by arrow (patient 3).

A 62‐year‐old male presented with generalized weakness and bony pains of 1‐year duration. He had difficulty walking for 3 months and used a wheelchair for 1 month. He had no history of any trauma. There was no history of similar illness in the family. He had received vitamin D supplementation before coming to our hospital. Systemic examination was unremarkable. His initial biochemical profile revealed persistent hypophosphatemia, decreased %TRP, and inappropriately normal serum FGF‐23 level. He had elevated serum intact parathyroid hormone levels (secondary hyperparathyroidism) for unknown reasons (Table 1, patient 3). In view of bony/muscular pains with persistent hypophosphatemia along with decreased %TRP, a diagnosis of TIO was made. Ga^68^ DOTANOC imaging revealed an expansile osteolytic lesion with an associated soft tissue component involving the angle and ramus of right mandible (20 × 20 × 12 mm in dimensions). Surgical removal of the lesion was discussed through intraoral route, which would involve removal of two teeth. The patient was not willing to have the surgery. Our interventional radiology team suggested RFA as a modality of treatment and the patient agreed for the same. The procedure involved the passage of electrode under CT guidance to the site of the lesion, followed by passage of electric current in the range of 200 to 1200 kHz to ablate the lesion (Fig. 2). At the time of RFA, a tissue sample was taken from the lesion for histological examination. Histology and immunohistochemistry suggested a diagnosis of phosphaturic mesenchymal tumor‐mixed connective tissue variant (PMT‐MCT). At 2‐weeks’ follow‐up, serum phosphate had normalized and the patient had remarkable clinical improvement. At last follow‐up of 10 months, his phosphorous was normal and he was able to walk without pain.

## Discussion

We describe 3 patients who had clinical and biochemical evidence of TIO. Ga^68^ DOTANOC imaging was successfully used to localize these small tumors in all 3 patients. Ga^68^ DOTANOC imaging is said to have the highest detection rate for these small tumors causing TIO.^7^ These tumors are typically benign, although rare cases of malignancy have been reported.^1^, ^9^ Complete excision of the tumor often cures the disease and is the standard of care. However, these tumors are usually present in surgically difficult locations where the need for less invasive modality is realized. This led to the consideration of other alternative treatment options. One emerging treatment option is minimally invasive, image‐guided tumor ablation. With this form of treatment, individual tumors are destroyed using heat (radiofrequency or laser ablation), cold (cryoablation),^10^ or chemical agents (percutaneous ethanol instillation). Ablative therapy is used as one of the preferred modalities of treatment in the management of soft tissue tumors such as osteoid osteoma, allowing rapid recovery with significantly shorter hospitalization.^11^ The goal of ablative therapy is complete tumor destruction. We used RFA for 3 patients with such tumors. It is based on the principle of achieving thermal ablation of the tissue by using alternating electric current that operates in the range of 200 to 1200 kHz. As the alternating electric current passes through the tissue, ions in the tissue attempt to follow the alternating direction of the current, frictional heat is generated that ablates the tissue in the vicinity of the electrode, and the conduction of the heat causes further tissue ablation.^12^ RFA is now a modality of choice in the management of benign bone tumors like osteoid osteoma, as RFA is associated with less duration of hospitalization and no major complications.^13^

There are only two reports, to the best of our knowledge, that have utilized RFA for treating patients with TIO (Table 2). Hesse and colleagues^14^ used two sessions of RFA in a 40‐year‐old woman with a lesion in the head of the femur. The success of the procedure was demonstrated by clinical improvement, phosphate normalization, and by MR imaging. Jadhav and colleagues^15^ subjected 3 patients with TIO to RFA. In their report, 2 patients achieved complete remission after a single session of RFA, while third patient continued to have persistent disease despite four sessions of RFA. His failure to achieve remission despite multiple sessions of RFA was mainly attributed to the bigger size of the lesion.

**Table 2:** Review of Similar Reports in the Literature

Parameters	Hesse et al., NEJM 2007^14^	Jadhav et al., JCEM 2014^15^		
Cases	Case 1	Case 1	Case 2	Case 3
Age (years)	40	38	28	49
Sex (M/F)	F	M	F	M
Duration of symptoms (years)	NA	6	8	17
Pre‐RFA serum phosphorus (mg/dL)	1.4	1.2	1.4	1.0
Post‐RFA (day 7) serum phosphorus (mg/dL)	NA	3.5	2.6	2.03
Pre‐RFA FGF‐23 (RU/mL)	NA	144.9	162.4	6000
Post‐RFA (day 7) FGF‐23 (RU/mL)	NA	23	41	5500
Pre‐RFA TMP/GFR (%)	0.8	1.25	0.68	0.25
Imaging	FDG PET‐CT	99mTc HYNIC TOC		
Site of lesion	Head, right femur	Head, right femur	Proximal shaft, left femur	Lower end, left femur
Size (mm)	NA	15 × 12	13 × 12	56 × 65
Histopathology	Benign mesenchymal tumor	NA	NA	Non‐ossifying fibroma
Duration of follow‐up after RFA (months)	12	12	15	6

RFA = radiofrequency ablation; FGF‐23 = fibroblast growth factor‐23; TMP/GFR = ratio of tubular maximum reabsorption of phosphate to glomerular filtration rate.

In our series, all 3 patients had small well‐defined lesions. Patients 1 and 3 were subjected to CT‐guided single sessions of RFA, whereas patient 2 was subjected to a USG‐guided single session of RFA. All patients required 1‐day hospitalization, and there were no adverse events. A limitation of our report is that we had no histopathological confirmation of the diagnosis in 2 patients (patients 1 and 2). However, we were able to demonstrate remission by clinical improvement, normalization of hypophosphatemia and hyperphosphaturia, and the disappearance of uptake on functional imaging (Ga^68^ DOTANOC PET/CT).

We have only up to 2 years’ follow‐up data of patients who underwent RFA or cryoablation for TIO in the literature, including our patients. Long term‐follow‐up data are needed to see whether the recurrence rate is different compared with surgical resection. In conclusion, although complete surgical resection is the standard of care, RFA can be an effective and safe alternative in patients with TIO, especially when resection is not possible because of inaccessible anatomical location, threat to vital nearby structures, or patient comorbidity that prohibits surgery.

## Disclosures

All authors state that they have no conflicts of interest.
